# Using implementation mapping for the adoption and implementation of *Target:BP* in community health centers

**DOI:** 10.3389/fpubh.2022.928148

**Published:** 2022-11-24

**Authors:** Melissa A. Valerio-Shewmaker, Natalia I. Heredia, Catherine Pulicken, Patenne D. Mathews, Roshanda Chenier, Tracy L. Swoboda, Ella R. Garza, Fernanda Velasco-Huerta, Maria E. Fernandez

**Affiliations:** ^1^University of Texas Health Science Center School of Public Health, Department of Health Promotion and Behavioral Science, Brownsville Regional Campus, Brownsville, TX, United States; ^2^Center for Health Promotion and Prevention Research, University of Texas Health Science Center at Houston School of Public Health, Houston, TX, United States; ^3^University of Texas Health Science Center at Houston School of Public Health, Department of Health Promotion and Behavioral Science, Houston, TX, United States; ^4^University of Texas Health Science Center at Houston School of Biomedical Informatics, Houston, TX, United States

**Keywords:** implementation mapping, *Target:BP*, hypertension, community health centers, evidence-based interventions, hypertension management

## Abstract

**Background:**

Despite the availability of multilevel evidence-based interventions for blood pressure management, poor hypertension control is common among community health center patient populations across the state of Texas and the United States. *Target:BP*^*TM*^ is a national initiative from the American Heart Association and the American Medical Association to assist healthcare organizations and care teams in improving blood pressure control rates using evidence-based approaches and recognition of organizations who have successfully integrated the program in their practice. Using the Implementation Mapping approach, we identified determinants of *Target:BP*^*TM*^ adoption and use and developed implementation strategies to improve program uptake and implementation in Community Health Centers in Texas.

**Methods:**

We used Implementation Mapping (IM) to identify barriers and facilitators influencing the adoption and implementation of the *Target:BP*^*TM*^ program and develop strategies to increase program adoption and use. We recruited four clinics across four counties in Texas and assessed barriers and facilitators at the organizational level, including electronic health records and data use. We used this data to inform clinic-specific implementation strategies based on the organization capacity and priorities feedback. We developed an implementation plan and timeline designed to improve the implementation and maintenance of *Target:BP*^*TM*^.

**Results:**

As part of the needs and capacity assessment, we collected data through interviews with CHC staff, examining gaps in needs and services (e.g., what do clinics need to implement *Target:BP*^*TM*^?), and assets to leverage. We worked with Community Health Centers to a) identify individuals who would be involved in the adoption, implementation, and maintenance of *Target:BP*^*TM*^, b) describe adoption and implementation actions, and c) identify barriers and facilitators influencing adoption and implementation. Together with partners from Community Health Center, we used the IM approach to identify and develop program goals, identify methods and strategies to address barriers, and create an implementation plan. Our strategies included monthly or biweekly meetings to provide technical support, reviewing program goals and timeline to ensure program implementation, progress toward reaching goals, and address quality improvement needs at each clinic site. We developed a *Target:BP*^*TM*^ implementation protocol for each clinic based on the needs and capacity assessment, identification of technology use and capacity, and gap analysis. We reviewed *Target:BP*^*TM*^ program strategies and self-measured blood pressure protocols tailored to the clinic patient population. We developed a collaborative plan, reviewed funding and capacity for implementation, and provided continuous quality improvement guidance. Ongoing process and impact evaluations using the Reach, Effectiveness, Adoption, Implementation, and Maintenance (RE-AIM) framework are underway.

**Discussion:**

This paper provides an example of using Implementation Mapping to develop strategies to increase the adoption and implementation of evidence-based cardiovascular risk reduction interventions in Community Health Centers. The use of implementation strategies can increase the use of *Target:BP*^*TM*^ in Community Health Centers and improve hypertension control.

## Introduction

Despite the availability of multilevel evidence-based interventions (EBI's) for blood pressure (BP) management, poor hypertension control is common among community health center (CHC) patient populations across the United States. It is estimated that almost 46.6% of the U.S. adult population aged 20 and over have high BP (i.e., systolic BP greater than 130 mmHg or diastolic BP greater than 80 mmHg) and/or are taking antihypertensive medications (1). Unscheduled physician and emergency room visits with hypertension as the primary diagnosis is of critical concern, with over 33.6 million health care and 1.1 million emergency room visits annually, costing over $131 billion each year (2). Moreover, half of all adults diagnosed with hypertension have uncontrolled hypertension and accounts for more than half a million deaths (12.7 deaths per 100,000 population) in the United States each year (3).

Considerable racial/ethnic, sex, and socio-economic disparities exist in hypertension diagnosis, treatment, and control. For example, Hispanic and Black males are disproportionally more likely to have hypertension than their female counterparts (4). Among adults with a diagnosis of hypertension, BP control is higher among non-Hispanic Whites (32%) compared with non-Hispanic Blacks (25%), non-Hispanic Asians (19%), and Hispanics (25%) (5). Further disparities are found by geographic regions, with Texas having a 32% prevalence of self-reported hypertension among adults compared to lower rates across the U.S. This reported prevalence may be an underestimation for Texas given the large uninsured and underinsured population; 18.4 percent of Texans were uninsured in 2019, double the national average, and numbers have risen due to the economic impact of COVID-19 and job losses (6).

Given these continuing health disparities, evidence based interventions addressing patient and organization level strategies to control BP in patients are highly needed. The *Target:BP*^*TM*^ program is a national initiative from the American Heart Association (AHA) and the American Medical Association (AMA) to assist healthcare organizations and care teams improve BP control rates through the implementation of evidence-based programming and recognition of organizations with successful integration. The unique aspect of *Target:BP* is the focus on building community clinic capacity to implement and maintain guideline-based care and promote accurate hypertension monitoring to improve patient-level outcomes. There are other EBIs that have been designed and implemented to address different aspects of hypertension control have been successfully implemented in community and clinic settings. For example, the *Million Hearts Collaboration* focuses on the alignment of cardiovascular disease prevention efforts through community linkages (7), the Healthy Heart Ambassador program supports community efforts through trained, certified ambassadors who provide one-on-one and group counseling to participants (8), and the WISEWOMAN program provides tools and resources to clinics that help women understand and reduce their risk of heart disease and stroke (9). Using the Implementation Mapping approach, we identified determinants of *Target:BP*^*TM*^ adoption and use them to develop implementation strategies to improve *Target:BP*^*TM*^ uptake and implementation in Texas CHCs, primarily Federally Qualified Health Centers (FQHCs) and look-alikes.

## Overview of *Target*:*BP*^*TM*^

The *Target:BP*^*TM*^ program is a national initiative formed by the AHA and the AMA to aid health care organizations to improve BP control through evidence-based quality improvement and clinical redesign. The program achieves this goal by helping practices ensure accurate BP measurement, empowering providers to start or increase treatment when BP is high at 2 or more office visits (10), and promoting shared decision-making and a patient-provider partnership to support patients' BP self-management through self-measured BP (SMBP), lifestyle changes and/or medication adherence, as appropriate (11).

The program provides participating clinics patient-facing materials on BP control to raise awareness, along with tools and resources for systems and process changes at the practice and/or health system level to improve BP management (12). The program promotes the use of 6 evidence- based activities to ensure accurate BP measurement (13): 1) calibrating BP measurement devices per manufacturer recommendations, 2) ensuring semi- and fully automated BP measurement devices are validated for clinical accuracy, 3) using a structured curriculum of at least 30 min every 6–12 months to increase staff knowledge and skills related to BP measurement, 4) using an objective skills demonstration assessment to test staff skills every 6–12 months, 5) using a BP measurement protocol to obtain consistent, accurate BP measurements, and 6) posting an infographic displaying best practices for accurately measuring BP in all locations where BP is measured.

For program recognition, practices are required to submit evaluation data (14), including their total adult patient population and breakdown by age, sex, ethnicity (15), those with hypertension, and those with controlled hypertension. Instructional videos and a data collection worksheet are provided to assist practices with collecting and submitting the evaluation data. The program recognizes organizations committed to improving BP control utilizing a tier system of recognition (Table 1) (16). Practices that achieve these successes are acknowledged by the AHA and AMA *via* various platforms (e.g., website, AMA and AHA national meetings) and provided with both promotional digital assets (e.g., digital seal for emails, social media messaging) and office items (e.g., plaque) to indicate achievement. While several of the activities recommended as part of the *Target:BP*^*TM*^ program can be considered implementation strategies themselves (training staff in BP measurement), the need to develop strategies to implement the *Target:BP*^*TM*^ program as a whole remained. Thus, we used Implementation Mapping for this purpose.

**Table 1:** Target:BP recognition levels based on evidence-based blood pressure activities completed.

**Recognition status**	**Activities required**	**Controlled hypertension rate**
Participation status	Submit data for the first time to the AHA Commits to reducing uncontrolled hypertension	–
Silver status	Submit data to AHA	–
	Complete 4/6 activities	
Gold status	Submit data to AHA	≥70%
	Complete 4/6 activities	
Gold+ status	Submit data to AHA	≥70%
	Complete 4/6 activities	

This table was created using the Target: BP levels of recognition for blood pressure control rates from Target: BP “Recognition Program” https://targetbp.org/recognition-program (accessed February 4, 2021). AHA, American heart association; BP, blood pressure.

## Implementation mapping

**Figure 1:**
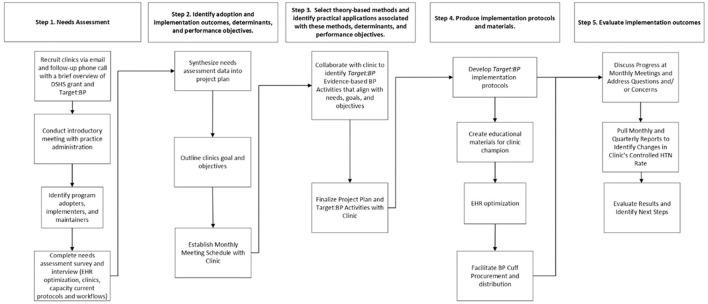
This workflow was created using the implementation mapping steps from Bartholomew Eldredge et al. (17). The adoption and implementation performance objectives examples influencing the adoption and implementation of the *Target*:*BP*^*TM*^ shown in this table came from the project planning. DSHS, Department of State Health Services; HER, Electronic Health Record; HTN, Hypertension; BP, Blood Pressure.

Implementation Mapping, a systematic process for developing or choosing implementation strategies is based on the Intervention Mapping, a protocol to guide the development of multi-level interventions (17). Specifically, Implementation Mapping expands on step 5 of Intervention Mapping (development of an implementation plan) and integrates both implementation science and health promotion to increase understanding of factors influencing implementation within a specific setting, and to guide the development of implementation strategies to increase intervention adoption, use, and sustainment (18). Implementation Mapping includes five tasks: 1) conduct an implementation needs and assets assessment and identify program implementers, 2) identify adoption and implementation outcomes, determinants, performance objectives (this includes the specific tasks or sub-behaviors required to carry out program adoption, implementation, and maintenance objectives), and develop matrices of change objectives (defined as the changes required for each determinant that will influence success of each performance objective), 3) select theory-based methods and identify practical applications associated with these methods, 4) produce implementation protocols and materials, and 5) evaluate implementation outcomes (18). For this project, we used an iterative process to identify barriers and facilitators influencing the adoption and implementation of the *Target:BP* program within the partner CHCs, and to develop a comprehensive plan for program integration (Figure 1).

## Methods

### Recruitment

We recruited four clinics from both rural and urban counties within Public Health Regions (PHR) 2/3 and 11, representing the greater Dallas-Fort Worth area and the Rio Grande Valley area of Texas, respectively. In collaboration with clinical leadership from the selected clinics and the research team, we used a team-based approach to promote and implement the *Target:BP* program. Our goal was to recruit a total of 5 clinics, a total of 20 clinics were contacted by email and telephone. Clinics were identified by location, previous history in partnership with the university, and by searching for Federally Qualified Health Centers serving the region. There were no financial incentives provided to the participating clinics.

As part of the recruitment, we completed discussions with the clinic office manager and or senior leadership to ensure decision making authority and need, priority, and interest in the program. The onboarding process differed at each clinic site. After the introductory meeting (i.e., within the following 2 weeks) the team and clinic partners completed the Needs Assessment survey and a program and organization capacity review. For each clinic, we first identified implementers and a program champion or primary contact to participate in the Implementation Mapping process to ensure each step was tailored to the clinic setting and the patient's needs. To recruit clinics, we followed a 4-step process: (1) identified clinics by email and follow-up phone call with a brief overview of the overall contract goals and *Target:BP*, (2) once agreed to serve as a site, we held an introductory meeting with clinic administration to learn about current practices, (3) conducted a needs assessment, and (4) synthesized information from the needs assessment to present to our clinic partner as part of our adoption/implementation step.

### Data collection

#### Electronic health record assessment

We began by assessing the clinic's EHR, hypertension practices, and collection of hypertension management data. Since utilization of EHR technology is an anchor for successful implementation of the *Target:BP* program, each clinic partner agreed to share EHR data and information with the UTHealth team. Thus, each clinic team collected data on-site and shared the data with the UTHealth team; the UTHealth team then reviewed and synthesized these data to inform discussions and assist each of the clinics with the EHR optimization needed to support and track the implementation of the *Target:BP* program.

#### Data collection and reporting

We used a standardized needs assessment instrument using both qualitative and quantitative measures to collect data at the start of the program. As part of this needs and capacity assessment, we conducted interviews with the staff at each clinic, examining gaps in needs and services (e.g., what do clinics need to implement *Target:BP*^*TM*^), and assets to leverage. Additional data and reporting occurred during the partner clinic's leadership and the research team monthly meetings to identify adoption and implementation outcomes while integrating strategies to enhance implementation of *Target:BP*^*TM*^ per CHC goals.

Following the limited data sharing agreements, each of the clinics provided the UTHealth team with quarterly data reports. Each of the clinics extracted data form the EHR and shared with UTHealth *via* secure email. The data reports helped us estimate baseline control hypertension rates and assess improvements over time as well as were included in funder reports. Additionally, as part of the Target:BP recognition program, each site submits annual data on hypertension rates and the activities completed the year prior.

## Results

For each CHC, we completed a needs assessment to determine organizational, patient, and capacity needs. We payed particular attention to barriers and facilitators at the organizational level to ensure the success and integration of changes within the CHC setting. We identified specific barriers and facilitators for adoption and implementation of *Target:BP*^*TM*^ including action steps for adoption and implementation of *Target:BP*^*TM*^ (e.g., who would complete what to implement) and determinants. Additionally, we developed and tailored clinic-specific implementation strategies which were informed by theory, empirical evidence, and organizational implementation team, including the program champion, leadership and others identified at the organization, feedback. Working in partnership with each of the four CHCs, we developed a tailored implementation plan and timeline designed to promote and enhance fidelity of implementation to promote maintenance of *Target:BP*^*TM*^ at each clinic.

### Task 1: Conduct a needs assessment to assess clinical capacity and identify program implementers

To guide the successful adoption of the *Target:BP* program, we had to understand each clinic's organizational capacity and identify program adopters, implementers, and maintainers. Thus, the first task involved conducting a thorough needs and capacity assessment. We initially contacted each of the selected CHC's leadership *via* email or telephone. During this initial contact, we provided the CHC with a general overview and scope of the *Target:BP* program as well as an introduction of the services our research team could provide to their clinics to facilitate the implementation of *Target:BP*. It is important to note that unlike many implementation research studies where participating clinics have previously agreed to implement a program, the approach described here included clinics who had not yet agreed to adopt or implement *Target:BP*.

Once the CHC was engaged and interested, an introductory virtual (e.g., Zoom or Microsoft Teams) meeting with the clinic's leadership was scheduled to present an overview of the *Target:BP* program (e.g., program participation levels, *Target:BP*^*TM*^ evidence-based activities for recognition, enrollment, and registration). During the virtual meeting, we also discussed clinical characteristics and practices and the patient population (e.g., type of organization, number of sites and providers, patient volume and sociodemographics, etc.). Clinics then identified and set goals for implementation of the program including EHR optimization, and hypertension management and prevention. We also used this opportunity to identify clinical staff and members of the clinical leadership team (i.e., Chief Operating Officer, IT/Data Analyst, Practice Administrator) who would be potential program adopters, implementers, and maintainers.

We then worked with the CHCs to identify their *Target:BP*^*TM*^ team, that is, the individuals who would be involved in the adoption and implementation of the *Target:BP*^*TM*^ program, to describe the adoption and implement actions, and identify barriers and facilitators from the needs assessment. Strategies were collaboratively developed to identify patient needs and program goals for their unique setting, and to develop methods and strategies to inform the implementation of *Target:BP*^*TM*^. We developed a *Target:BP*^*TM*^ implementation protocol based on the needs assessment for each clinic based on the needs and capacity assessment, identification of technology capacity and use, and gap analysis findings. We reviewed *Target:BP*^*TM*^ program strategies and SMBP protocols tailored to the clinic patient population. Details on clinic characteristics and identified patients' needs were used to address the multi-level needs for dissemination and implementation of this evidence-based program (Table 2).

**Table 2:** Results from community health centers needs assessment survey.

	**Community health centers**			
**Characteristics**	**CHC A**	**CHC B**	**CHC C**	**CHC D**
PHR region	11	2/3	2/3	2/3
Type of clinic	FQHC	FQHC	FQHC	CHC
**Patient demographics**				
***Race and ethnicity***				
Hispanic or latino	73.00%	40.80%	73.83%	0.00%
Non-hispanic or latino	27.00%	59.20%	26.17%	100%
Native american or alaskan native	0.05%	0.19%	0.00%	92.53%
Asian	0.05%	0.67%	20.48%	0.00%
Black	0.38%	17.77%	41.65%	0.00%
Native hawaiian/other pacific islander	0.04%	0.35%	0.00%	0.00%
White	13.76%	61.38%	91.03%	
Other (not indicated)	0.49%	19.63%	0.00%	7.47%
***Type of insurance coverage***				
Private insurance	14.55%	15.29%	5.28%	17.05%
Medicare	7.70%	9.25%	1.99%	11.12%
Medicaid	23.92%	23.46%	20.73%	3.73%
Uninsured/self-pay	52.21%	51.11%	72.01%	50.53%
Other (Not Indicated)	0.34%	0.89%	0.48%	0.00%
***Hypertension management qualitative indicators***				
CHC awareness of *Target:BP^*TM*^* prior to implementation	Yes	Yes	Yes	Yes
CHC use of CDS tools in EHR to identify, treat, and manage patients with hypertension	Yes	Yes	Yes	No
CHC use of SMBP protocol for patients diagnosed with hypertension	Yes	Yes	No	Yes

The data displayed in this table come from the Community Health Centers Needs Assessment Survey results completed as part of the Implementation Mapping process. PHR, Public Health Region; CHC, Community Health Center; CDS, Clinical Decision Support; HER, Electronic Health Record; SMBP, Self-Measured Blood Pressure; BP, Blood Pressure.

The next steps included completion of a Memorandum of Understanding (MOU), needs assessment, project plan development, and program delivery. After completion of the MOU between the designated CHC and UTHealth, a needs assessment survey was administered *via* Qualtrics to determine the CHC's hypertension workflow, the use of clinical decision support (CDS) tools for hypertension-related practices (i.e., patient identification, treatment, and management), recommendation of evidence-based activities (i.e., self-measured BP monitoring [SMBP]), patient portal usage, as well as hypertension outcomes related to BP control.

Identifying the CHC's implementation capacity was a critical step since many times, the CHC's staff may not have time to assess and identify all variables needed for the implementation of an EBI. These tasks were completed in collaboration with the CHC and the UTHealth team, which allowed for real-time data sharing to inform the tailoring of the *Target:BP* program implementation. Following these first steps, an in-depth interview was scheduled with clinical leadership to further assess current workflows and data reporting, and identify the CHC's barriers and facilitators for the implementation of *Target:BP*. After completion of the needs assessment, a follow-up meeting was scheduled with the CHC's stakeholders to discuss results, identify areas for improvement and initiate a project implementation plan.

As noted above, the initial meeting with each of the clinics and follow-up meetings were conducted by videoconference, and the scheduling and coordination were completed by email and based on the availability of the clinic sites. Communication with each of the clinics related to activities and goals between program implementation meetings was completed by email and phone calls. Specifically, UTHealth scheduled and provided support to each of the clinics and the program consultants to ensure coordination and facilitation of meetings and focus on activities, partnerships and goals. At each clinic site, attendance at meetings usually included the clinic's leadership, management team and implementers including medical directors, nurse team members, patient navigators, operation managers and information technology team members.

### Task 2: Identify adoption and implementation outcomes, determinants, and performance objectives, and develop matrices of change objectives

To facilitate the development of a project implementation plan that aligned with each CHC's hypertension management goals, the UTHealth team created a project planning guide to identify gaps in hypertension management and address specific priorities and tasks for program implementation. In collaboration with each of the CHC's program adopters and implementers, the project plan was finalized by the UTHealth research team and the CHC's leadership. The planning team identified adoption and implementation outcomes for adopters, implementers and maintainers (e.g., clinic's leadership, providers, administrative staff, non-physician team members [community health workers, physicians' assistants, etc.]) for each CHC see implementation outcomes for each (Table 3). The team worked together in the creation of performance objectives to identify “who needs to do what to ensure that the program is adopted, implemented, and maintained?” For example, the *CHC's leadership (decision-makers) agree to participate in the Target BP program by enrolling in the Target:BP program with an AHA representative*. These performance objectives served as a roadmap essential for the adoption, implementation, and maintenance of the *Target:BP* program.

**Table 3:** Example of Implementation outcomes and performance objectives.

**Program: Target:BP**		
**Setting: Community Health Center (CHC)**		
**Target: Role**	**Adoption, implementation and maintenance outcome**	**Performance objectives**
**Adopter:** CHC leadership	CHC leadership will adopt the Target: BP program and associated recommendations within their practice to improve hypertension control among at-risk patient population.	1. Establish/re-engage with AHA representative in selected public health region to register and plan implementation. 2. Designate a program champion and a point of contact to review Target: BP program and lead the implementation. 3. Agree, approve, and support the adoption of the Target: BP program. 4. Establish and sign MOU with AHA and UTHealth. 5. Assess that CHC is equipped with sufficient materials and equipment for identification of hypertensive patients and program implementation. 6. Approve steps and assure funding and practice of the Target: BP program.
**Implementer:** Program champion	The provider and program champion will implement the Target: BP program into their hypertension management protocol.	1. Enroll and work with AHA representative in selected public health region to complete online registration and begin implementation of Target: BP program. 2. Obtain and distribute program materials focused on hypertension management and protocol recommendations for providers and patients. 3. Establish effective communication among CHC staff and ensure updates and feedback is delivered in a consistent manner. 4. Identify barriers and communicate suggestions for overcoming them. 5. Provide continuous support for decision making (feedback, quality check and monitoring consistency of delivery) and provide monthly reporting on program adoption and patient outcomes. 6. Report on Target:BP program adoption and patient outcomes once a month.
**Maintainer:** Program champion	Program champion will maintain the Target BP program and ensure the successful delivery of program resources and materials to the designated CHC staff.	1. Discuss the integration of the Target: BP program with leadership and with the implementation team. 2. Maintain supply of resources and program materials, as well as any needed changes in any program materials given the CHC setting and patient population (health management action plan review) 3. Use EHR to maintain Target: BP patient outcome goals and ensure CHC hypertension evaluation data is submitted in a timely manner to the Target: BP program.

The implementation outcomes and performance objectives for adopters, implementers and maintainers displayed in this table were established by the planning team for the adoption and implementation of Target:BP program. CHC, Community Health Center; AHA, American Heart Association; BP, Blood Pressure.

After the identification of program outcomes and performance objectives for each of the CHC's stakeholder groups, we defined determinants for *Target:BP* program implementation (Table 4).

**Table 4:** Example of partial matrices of change objectives for selected examples.

**Program: Target:BP**					
**Behavioral outcome: Implement the Target:BP program into hypertension management protocol**.					
**Performance objectives**	**Determinants**				
	**Attitude**	**Knowledge**	**Outcome expectations**	**Self-efficacy/skills**	**Social norms**
Program champion enrolls and works with AHA representative in selected public health region to complete online registration and begin implementation of Target: BP program.	A1.1. Express positive attitude towards the implementation of Target BP.	K. 1.1. Describes the components of the Target BP program. K.1.2. Describes the rates of uncontrolled BP in CHC. K.1.3. Describes requirements of the Target BP program	OE.1.1. Expects that by attending the training he/she will be able to successfully implement Target BP OE.1.2. Expects program champion and CHC leadership will reinforce and acknowledge them for completing the training successfully	SSE.1.1. Feels confident in ability to attend and learn from training. SSE1.2. Expresses confidence in attending Target BP training SSE.1.3. Expresses confidence in the ability to do what is expected by the Target BP (increase screening capacity, implementation of the program, work with a program champion, assess resources)	NB1.1 Expresses belief that other CHC like theirs are implementing Target BP NB1.2. Expresses belief that other coordinators attend training.
Program champion obtains and distributes program materials focused on hypertension management and protocol recommendations for providers and patients.	A.2.1. Expresses that Target BP program information will help patients with BP management.	K.2.1. Describes the role of each CHC team member for implementation. K.2.2. Describes patient education needs. K.2.3. Describes toolkits and other materials that support program implementation given specific staff role.	OE.2.1. Expects that by providing staff and patients with information Target BP uptake will be achieved. OE.2.2. Expects that patients will use Target BP information for BP control management	SSE.2.1. Feels confident in identifying Target BP components to share with specific team members based on role in CHC. SSE.2.2. Feels confident in identifying Target BP materials to share with the patient population.	NB2.1. Expresses belief that other coordinators are identifying Target BP components for staff and patients.
Program champion develops strategies to identify upcoming appointments for patients with uncontrolled blood pressure daily.	A.3.1. Believes in the importance of identifying upcoming appointments.	K3.1. Describe steps to searching schedule to identify upcoming appointments. K.3.2. Describes the data system of the CHC A.3.3. Describes process of using data systems to identify upcoming appointments.	OE.3. Expect that all scheduled patients will be identified for receiving Target BP program information.	SSE.3.1. Express confidence in and demonstrates ability to successfully identify all upcoming appointments	NB3. Express belief that other program coordinators are searching schedules for upcoming appointments.
Program champion oversee implementation efforts and provide feedback to CHC staff	A.4. Feels positive about overseeing implementation as important and useful for ensuring fidelity	K.4. Describes steps needed to oversee implementation. K.4.1. Describes daily and weekly activities associated with Champion Role.	O.E.4. Expects that through regular oversight and communication, the Target BP program will be implemented effectively.	SSE.4. Demonstrates confidence and ability to oversee implementation of Target BP.	NB.4. Believes that other individuals with similar positions in other CHCs act as champions to oversee and provide feedback.
Program champion identify barriers and communicate suggestions for overcoming them.	A.5. Recognizes that identifying barriers is important to the success of the project.	K.5. Lists potential barriers to implementation and solutions that could address them.	O.E. 5. Expects that the early identification of barriers to implementation will lead to effective solutions that will facilitate continued program use.	SSE.5. Expresses confidence and demonstrates the ability to identify problems during implementation and to work with other implementers to resolve them.	NB. 5. Believes that other champions like them have a role that includes the identification and resolution of barriers.

The performance objectives displayed in this table were established by the planning team for the adoption and implementation of Target:BP program using theoretical constructs from the social cognitive theory.

CHC, Community Health Center; AHA, American Heart Association; BP, Blood Pressure.

These determinants are derived from theoretical constructs that aligned with the barriers and facilitators identified in the needs assessment. The UTHealth team completed a thorough literature review related to *Target:BP* program implementation and similar evidence-based BP control programs. At this point, we used literature to inform the identification of the priority determinants given the results from each of the CHC's needs assessment, allowing us to identify potential determinants that could impact the CHC stakeholder group's ability to achieve their outcomes. Once determinants were reviewed by the teams, we created the change matrices. The matrices of change objectives for each CHC's stakeholder group list the various changes in each determinant necessary to achieve the associated performance objective. The use of these matrices helped ensure that content and messaging for the implementation of *Target:BP* addressed the most salient performance objectives and determinants to facilitate successful implementation.

While each clinic implemented the program based on their own goals and capacity, the team recognized some key factors that promoted implementation including the collaboration of change agents, such as the clinic site leadership team and stakeholders that were aware of the community and organization capacity as well as could identified potential resources.

### Task 3: Select theory-based methods and associated practical applications

After defining the necessary changes needed within the CHCs for successful *Target:BP* program implementation, we then identified evidence- and theory-based strategies to address these changes at the provider and administrative levels, and developed tables highlighting methods and practical applications (Table 3). These strategies addressed determinants identified using theoretical constructs from the social cognitive theory and organizational level frameworks. We reviewed the behavioral and implementation science literature to ensure that the appropriate methods were identified to facilitate change and address determinants and change objectives for each CHC. For *Target:BP*, these methods and applications were developed from existing materials, messages, and recommended practices. For each of the recommended practices and steps of *Target:BP*, we identified how their implementation would address determinants and change objectives. This facilitated CHCs' staff training, identification of materials needed for *Target:BP* implementation, and strategies to gain access to the materials needed. For example, in one CHC we found that while training addressed knowledge, self- efficacy and perceived norms for BP monitoring and capacity, the CHC did not have the proper equipment to implement the *Target:BP* protocols at the patient level. Specifically, many of the CHCs could not afford the BP cuffs for the necessary patient population and cuffs were not available in all the needed sizes (e.g., XL BP cuffs). However, we were able to work with other partners (i.e., AHA) to identify potential sources for the equipment at discounted prices. Implementation Mapping facilitated the review of contextual factors that influenced implementation and allowed for CHCs to identify resources and other actions needed to properly implement *Target:BP*^*TM*^.

### Task 4: Produce protocols and materials related to implementation

Working together with the CHC's leadership and implementation team members, we developed protocols and activities needed for the implementation of key *Target:BP* objectives. These activities included training and re-training of CHC's staff on proper BP techniques, EHR optimization, and the development of tailored *Target:BP* materials. While *Target:BP* materials may be readily available for adaptation through the AHA (i.e., targetbp.org), we worked with the CHCs to ensure proper wording and design of certain materials (e.g., flyers on proper BP techniques) to target the CHC's hypertension management goals, clinical setting, and patient population. To ensure the appropriate selection of the intended audience, target determinants, change objectives, and material content, we closely collaborated with the CHCs in the development of protocols, workflows, and materials used for the implementation of *Target:BP*. The workflows identified potential adopters, implementers, and maintainers and visually depicted how *Target:BP* would be integrated into the CHC's current or new hypertension care management process. These workflows were then communicated and used to guide *Target:BP* implementation at the clinic. We carefully reviewed with the CHCs to ensure future uptake and dissemination and promote adoption and use as well as to help with future implementation and impact evaluation.

### Task 5: Evaluate implementation outcomes

**Figure 2:**
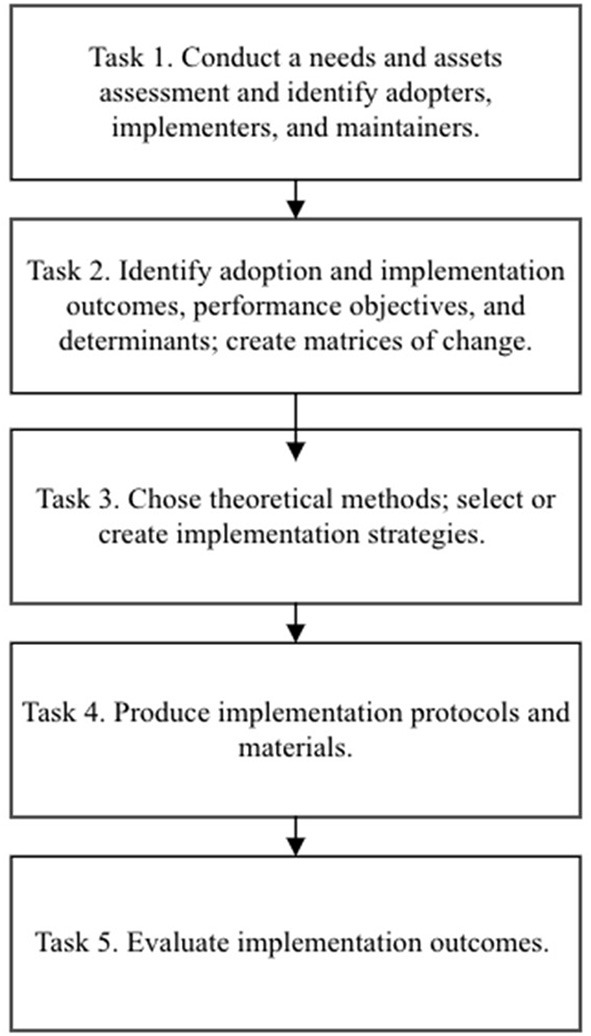
Implementation mapping process from Fernandez et al. (18).

Ongoing evaluation of implementation outcomes for *Target:BP* use within CHCs has identified several key areas to improve reach, engagement, and impact, including the integration of key clinic specific team members and community partners to promote use of data and inform strategies to implement at each clinic. The collection of process data including reach of patients in most need will allow the team to identify the impact of implementation strategies as well as essential preconditions and changes at the CHC level that facilitated implementation, fidelity, and reach of the patient population. We expect that CHC organizational process evaluation and impact data will allow us to better identify barriers and enabling factors for *Target:BP* adoption, implementation, and sustainability outcomes. Once we complete the evaluation, we will use findings to improve *Target:BP* delivery and for interpreting patient-level outcomes. We will be able to better identify whom the program reached, assess fidelity of implementation, and determine organizational factors that influence intervention, adoption, use, and or maintenance (Figure 2).

## Discussion

The implementation of the *Target:BP* program provides an example of the use of Implementation Mapping for the development and adoption of evidence-based strategies to increase successful implementation of evidence-based programs within CHCs. The use of implementation strategies can increase the use of *Target:BP*^*TM*^ in CHCs (organizational level) and improve hypertension control outcomes (patient level). The steps of Implementation Mapping allowed us to carefully integrate and address the specific needs of CHCs at their pace while addressing the importance of fidelity and reach of not only patients, but also adopters and implementers, to ensure success.

Team meetings ensured that both the CHC's leadership and the UTHealth team listened to implementer needs and facilitated changes in information technology (IT), EHR, and training. This fostered the development of successful training to address the needs of facilitators as well as strategies to overcome adoption and implementation barriers encountered by the CHC teams including IT, managers, nurses, and other health care providers. The *Target:BP* implementation at CHCs allowed the research team to test the conceptual and practical gaps between identifying barriers and facilitators, and developing strategies for immediate communication and problem solving to strengthen and increase the ease for adoption and implementation of *Target:BP*. The CHCs identified and addressed changes in implementation to contextual factors that allow for greater learning, openness, and identification of CHC setting needs to impact health and quality of life of patients.

Given the ongoing challenges to implement EBIs successfully, the use of Implementation Mapping may help (a) increase the confidence, capacity, and readiness of CHCs to use EBIs by elucidating mechanisms for change within their CHC, (b) inform the planning process to ensure the identification of determinants of change, and (c) select implementation strategies with the greatest potential for impact on health outcomes over time.

As this is an ongoing program, we expect that our iterative approach to Implementation Mapping across additional CHCs will allow us to reach and expand our knowledge of the use of Implementation Mapping as a planning framework for the successful delivery of EBIs aimed to improve health. Ongoing process and impact evaluations using the Reach, Effectiveness, Adoption, Implementation, and Maintenance framework (RE-AIM) are underway to evaluate the *Target:BP*^*TM*^ program (19).

It is well documented that EBIs may not be adapted or adopted in settings that may most benefit from their impact (20–23). However, Implementation Mapping outlines a practical step-by-step method for planning of implementation to optimize reach, appropriateness, and impact over time, and that simultaneously will build capacity at CHCs and similar settings to adopt, implement and sustain evidence- and guidelines-based practices to improve health outcomes.

The CHCs implementing the *Target:BP*^*TM*^ program will have tools to ensure maintenance and reach of patients with the most need.

## Data availability statement

The raw data supporting the conclusions of this article will be made available by the authors, without undue reservation.

## Ethics statement

The studies involving human participants were reviewed and approved by University of Texas Health Science Center Houston, Institutional Review Board. Written informed consent for participation was not required for this study in accordance with the national legislation and the institutional requirements.

## Author contributions

All authors listed have made a substantial, direct, and intellectual contribution to the work and approved it for publication.

## Funding

This publication was supported by the Centers for Disease Control and Prevention of the U.S. Department of Health and Human Services (HHS).

## Conflict of interest

The authors declare that the research was conducted in the absence of any commercial or financial relationships that could be construed as a potential conflict of interest.

## Publisher's note

All claims expressed in this article are solely those of the authors and do not necessarily represent those of their affiliated organizations, or those of the publisher, the editors and the reviewers. Any product that may be evaluated in this article, or claim that may be made by its manufacturer, is not guaranteed or endorsed by the publisher.

## Author disclaimer

The contents are those of the author(s) and do not necessarily represent the official views of, nor an endorsement, by Texas Department of State Health Services (DSHS), CDC/HHS, or the U.S. Government.
